# A new approach to stabilize waste biomass for valorization using an oxidative process at 90 °C

**DOI:** 10.1371/journal.pone.0196249

**Published:** 2018-04-23

**Authors:** Takanori Itoh, Kazunori Iwabuchi, Kumpei Ota

**Affiliations:** 1 Graduate School of Agriculture, Hokkaido University, Sapporo, Hokkaido, Japan; 2 Research Faculty of Agriculture, Hokkaido University, Sapporo, Hokkaido, Japan; Universita degli Studi della Tuscia, ITALY

## Abstract

This study aimed to establish a new methodology for upgrading biomass quality using low-temperature (below 100 °C) oxidation to achieve simultaneous drying and decomposition. Sterilized manure (63% wet basis) was heated at 90 °C for 49 days under an oxidative environment. The obtained solid and moisture reduction curves indicated that drying and decomposition proceeded simultaneously. The biomass was decomposed by oxidation with the release of water, carbon dioxide, and volatile fatty acids such as acetic acid. The oxidation process stopped when the biomass was dehydrated, indicating that the water originally present in the biomass governed the process. Elemental and calorific analyses revealed no remarkable increase in carbon content or increased heating value, and a slight decrease in oxygen content. Although the severity of the process was insufficient to produce an optimum solid fuel due to the low temperature used, the process would enable the stabilization of waste biomass with low energy consumption such as using waste heat.

## Introduction

Biomass is an alternative energy source to fossil fuels that can also be converted into fuels and chemicals. Consequently, there is increasing interest in biomass conversion technologies to mitigate climate change. Several types of waste biomass, such as livestock manure, sewage sludge, and food waste are produced globally in large quantities and continuously. Due to the many impurities in waste biomass, it is more practical to convert it into biofuels rather than into fine chemicals. Approximately 43 EJ (1 EJ = 1 × 10^18^ joule) of livestock waste is produced annually [1], which corresponds to about 72% of the demand in bioenergy for 2014 [2] and is estimated to increase as the number of livestock increase to meet food demand. Thus, waste biomass has huge potential as an energy source and its utilization will promote sustainable waste management and mitigate environmental pollution.

Currently, only 5.4 EJ of livestock waste is used annually for energy production [1], highlighting the difficulty in converting waste to energy due to the high moisture, hydrophilic nature, and low transportability of waste biomass. Consequently, biomass is usually subjected to a single or multiple pretreatments to aid its use in energy production. Dry or wet torrefaction of waste biomass generates solid biofuels called biochar and hydrochar, respectively [3,4]. Chemically, both techniques remove oxygen in the raw biomass, producing carbon-rich materials. In dry torrefaction, biomass is heated at 200–300 °C under an inert or oxygen-limited environment, causing thermal degradation [5]. For example, Brachi et al. demonstrated that wet agro-industrial residue (e.g., sugar beet pulp and tomato peel residues) was converted into high-quality solid fuel through dry torrefaction [6,7]. However, drying is necessary and is the most energy-intensive step in dry torrefaction, thereby greatly affecting its production efficiency [8]. Consequently, waste biomass with a high moisture content is rarely used in dry torrefaction. Wet torrefaction is promising for waste biomass conversion because the moisture content has little effect on the process, which involves using hot compressed water at temperatures of 180–260 °C [4,9,10]. However, the generation of hot compressed water requires high-pressure equipment, increasing the investment and operating costs of the process on an industrial scale [4]. In addition, drying wet hydrochar for subsequent use in pyrolysis, combustion, or gasification is problematic [4]. For these reasons, dry torrefaction appears to be more practical for commercialization.

The current study aimed to extend our understanding of the thermochemical conversion of waste biomass for use in dry torrefaction by combining the two steps of drying and torrefaction (decomposition) into one, thereby reducing the energy required for biomass conversion. Since the temperature of wet biomass never exceeds the boiling point of water, biomass decomposition should be carried out at temperatures below 100 °C and we hypothesized that oxidation occurring below 100 °C would both achieve decomposition and improve biomass quality. Biomass oxidation is typically inefficient at such low temperatures but under certain limited conditions, low-temperature oxidation proceeds when wet biomass is exposed to oxygen [11–14] because water promotes the chemical adsorption of oxygen, resulting in a chemical reaction between oxygen and the biomass. Oxygen adsorption results in the generation of unstable intermediates that eventually decompose into CO, CO_2_, H_2_O, and volatile fatty acids (VFAs) [15,16]. These decomposed products contain oxygen, suggesting that low-temperature oxidation should both remove oxygen and enhance the relative carbon content of biomass. Thus, the objectives of the current study were to: (1) simultaneously dry and decompose biomass by oxidation at 90 °C, and then (2) evaluate the proposed methodology by analyzing the quality of the biomass produced.

## Materials and methods

### Materials

Dairy cattle manure from an experimental farm (Field Science Center for Northern Biosphere, Hokkaido University, Japan, latitude 43° 04′ N, longitude 141° 20′ E) was used. We eliminated the involvement of microbes during the process by sterilizing the raw manure in an autoclave at 121 °C for 15 minutes. Prior to the experiments, the moisture and ash content were determined by drying a wet sample at 105 °C for 24 hours using a laboratory electric oven to measure the moisture content and incinerating 2 g of the oven-dried sample at 600 °C for 3 hours using a muffle furnace to determine the ash content. To fix the ventilation rate (L min^−1^ kg-AFS^−1^), the total amount of organic matter (volatile matter (VM) and fixed carbon (FC)) was expressed as ash-free-solid (AFS), determined by subtracting the ash from the dry solid.

### Experimental procedure

**Fig 1:**
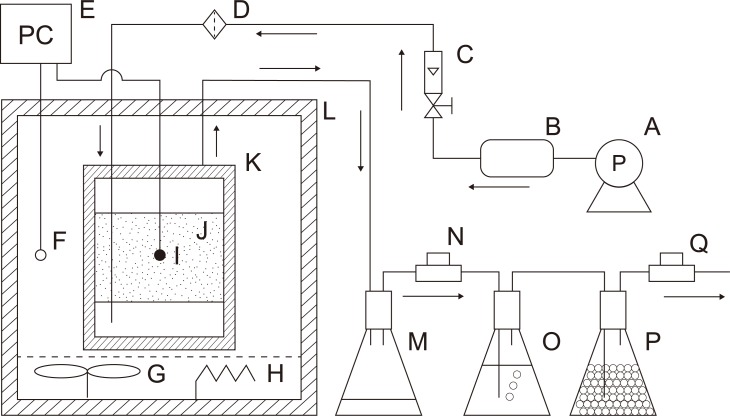
Experimental setup of the reaction system. A: air compressor (replaced by a nitrogen cylinder for inert conditions); B: air receiver tank; C: flowmeter; D: sterilized filter; E: PC; F: chamber temperature; G: fan; H: heater; I: sample temperature; J: sample; K: reaction vessel; L: adiabatic chamber; M: liquid collection; N: gas sampling port; O: ammonia trap; P: silica gel; Q: oxygen sensor.

A schematic of the reaction system is shown in Fig 1. The reaction vessel containing the sample (about 200 g wet mass, 63% wet basis (wb)) was heated at 90 °C in an adiabatic chamber. The air (oxygen concentration 21 vol.%) was supplied from the bottom to the top of the vessel by an air compressor. The ventilation rate was set at 0.025 L min^−1^ kg-AFS^−1^ and was regulated by a flowmeter (Model 1200 series flowmeter with precision needle valve, Kojima Instruments, Inc.). This ventilation rate supplied sufficient oxygen for oxidation to occur while maintaining the water content of the sample as long as possible during the process. A sterilized filter (pore size: 0.20 μm) was inserted at the inlet to prevent microbes from entering the reaction vessel. For comparison, nitrogen gas was also supplied from a nitrogen cylinder under the same conditions in separate experiments.

The condensate liquid, containing evaporated water, was collected in a bottle positioned at the outlet. The mass and pH of the condensate liquid were measured weekly. The VFAs collected in the condensate liquid were identified using a gas chromatograph-mass spectrometer (JMS-Q1000GC, Jeol Ltd.). The amounts of CO and CO_2_ were measured daily using a gas chromatograph (GC-4000, GL Science, Inc.). The O_2_ concentration was monitored every 5 minutes with an O_2_ sensor (New Cosmos Electric Co., Ltd.). Each gas concentration was converted to the uptake or production rate by following a previously reported procedure [17].

The degradation coefficient *K* was used to evaluate the residual amount of AFSs in the samples during the process. Assuming *K* g of material decomposed when 1 g of CO_2_ was produced, *K* was calculated as

$$K=\frac{\int \Delta \mathrm{AFSd}t}{\int \Delta {\mathrm{CO}}_{2}dt}$$ (1)

where *K* is the degradation coefficient [g-AFS g-CO_2_^−1^], ΔAFS is the AFS loss [g], and ΔCO_2_ is the amount of carbon dioxide produced [g]. Solid reduction curves were prepared based on the degradation coefficient, amount of emitted CO_2_, and initial amount of AFS. Moisture reduction curves were prepared based on the amount of trapped water and the initial amount of water in the manure.

### Sample analysis

The amounts of C, H and N in the samples were detected using an elemental analyzer (CE-440, Exeter Analytical, Inc.) and the oxygen content was calculated by subtraction (O = 100 − C − H − N − Ash). The reduction rate of each element was determined by the ash tracer method [18]. This method assumes that biomass ash is conserved within the treated biomass and the ash fraction in biomass is not affected by the process. Higher heating values (HHV) were measured with a bomb calorimeter (O. S. K 200, Ogawa Sampling Co. Ltd.).

## Results

### Changes in the appearance of the manure heated at 90 °C

**Fig 2:**
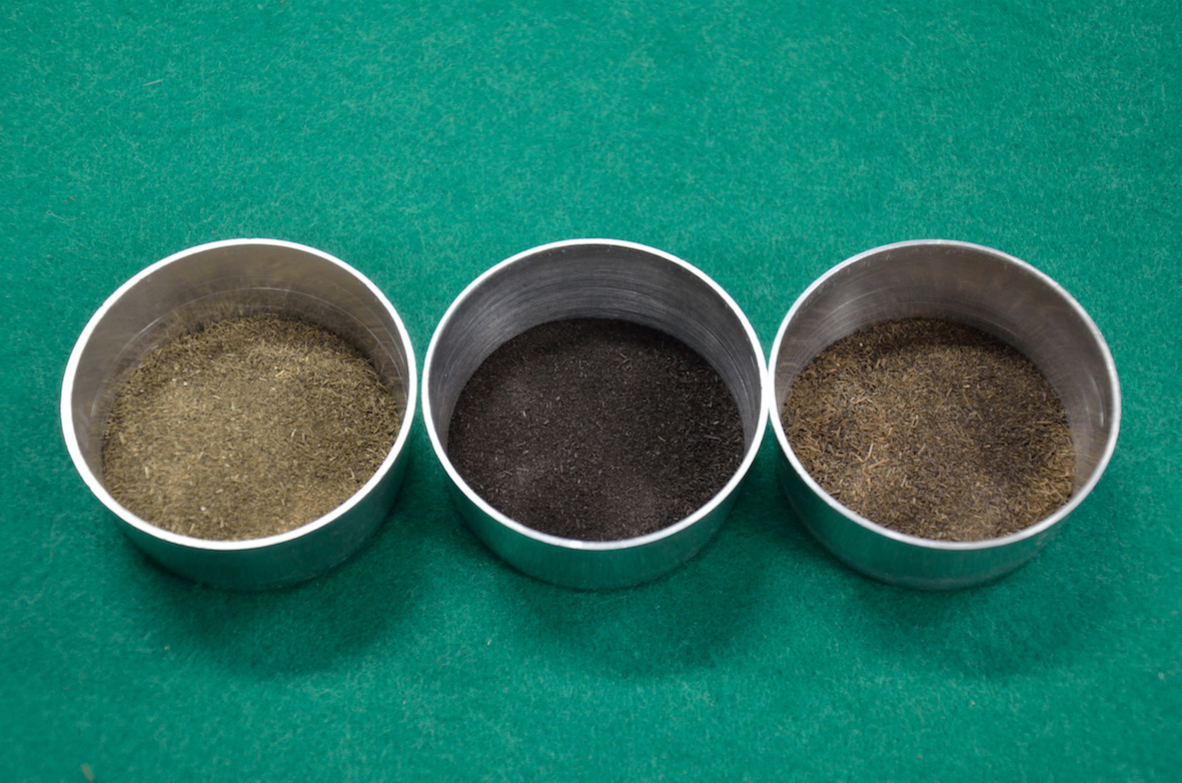
**Appearance of raw dairy manure (left), manure treated under air at 90 °C (center), and manure treated under N_2_ at 90 °C (right).** All samples were dried before being photographed to eliminate the effect of water on the color.

The appearance of sterilized manure heated at 90 °C either in air or under nitrogen is shown in Fig 2. Manure heated under an air environment became darker whereas manure heated under a nitrogen environment did not change significantly in color. The change in biomass color is a simple indicator of a chemical reaction related to low-temperature oxidation.

### Determination of the gaseous and volatile species during biomass decomposition

**Fig 3:**
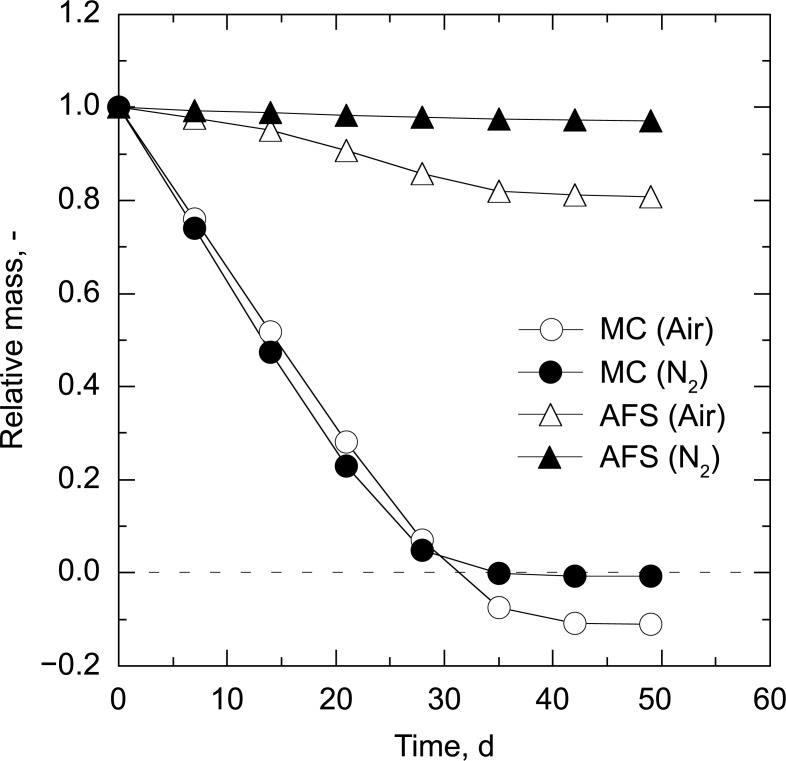
Solid and moisture reduction curves.

**Fig 4:**
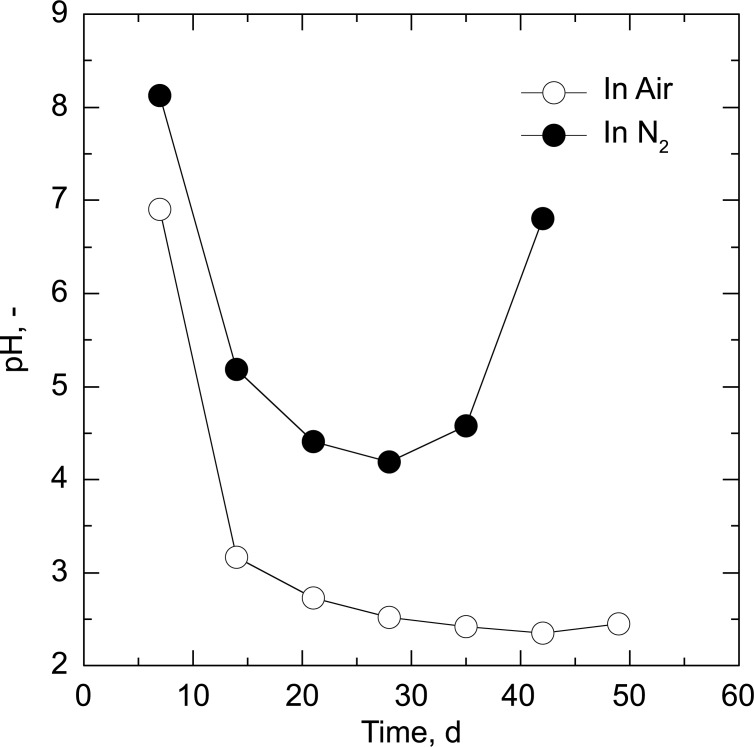
pH values of the condensate liquid.

**Fig 5:**
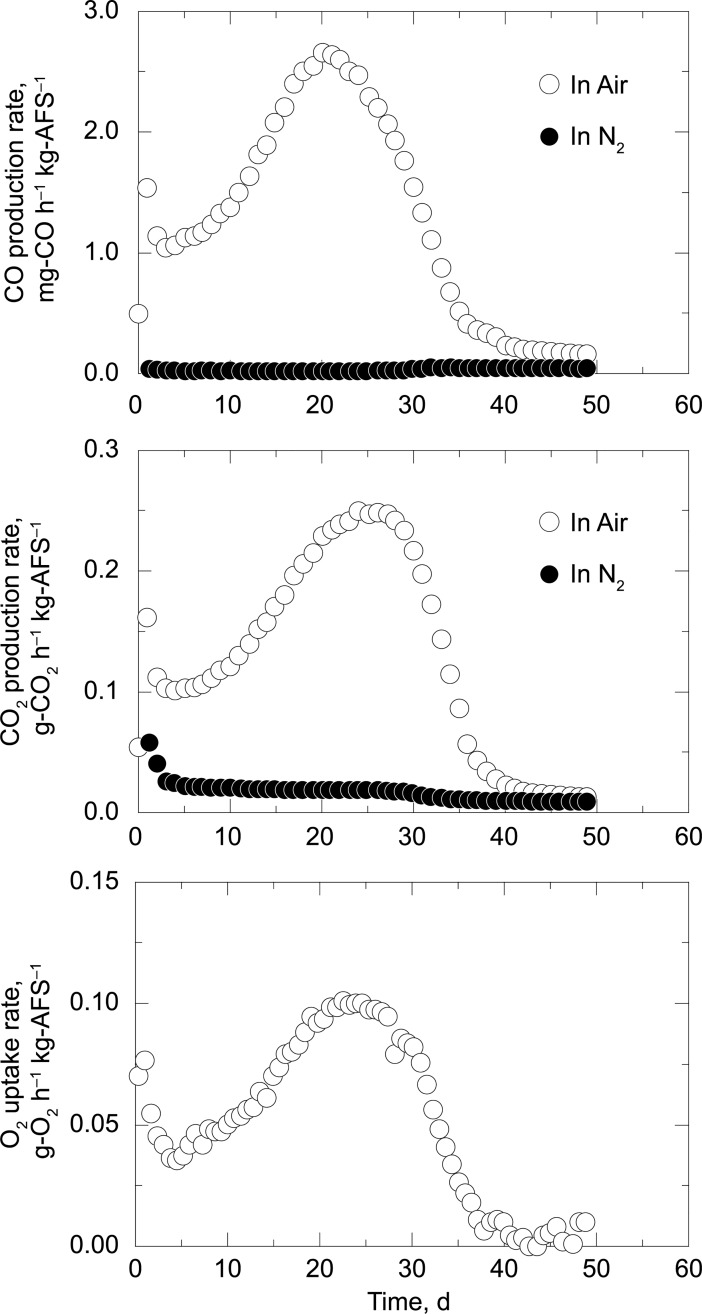
CO and CO_2_ production rates and O_2_ uptake rate.

The solid and moisture reduction curves are shown in Fig 3, confirming that decomposition and drying of the manure proceeded simultaneously in air, and that decomposition stopped when the water content of the manure became very low (less than 1.0%; Table 1). The moisture reduction curve dropped to negative values when the experiments were carried out under an oxidative environment, strongly suggesting that water and water-soluble compounds were produced during the decomposition process. Indeed, the pH of the condensate liquids was strongly acidic (pH lower than 3) due to the generation of VFAs such as acetic, propionic isobutyric, and isovaleric acids (Fig 4 and S1 Fig). CO and CO_2_ evolution was accompanied by O_2_ consumption (Fig 5). The maximum rate of gas evolution was observed at the point when the constant drying rate was changed (critical moisture content) at day of 28 (Figs 3 and 5). In contrast, under a nitrogen environment, neither biomass decomposition nor CO and CO_2_ evolution were observed, and only the drying process proceeded.

**Table 1:** Physicochemical properties of the raw manure and manure treated at 90 °C (wb: Wet basis; db: Dry basis).

	Moisture content	Ultimate analysis (wt.%db)					Atomic ratio		HHV(db)
	wt.%wb	C	H	N	O	Ash	O/C	H/C	MJ/kg
**Raw manure**	63.33	43.36	5.56	2.27	35.44	13.38	0.61	1.54	17.75
**Treated manure**	0.90	43.11	4.46	3.39	30.69	18.35	0.53	1.24	16.93

### Physicochemical properties of the product

**Fig 6:**
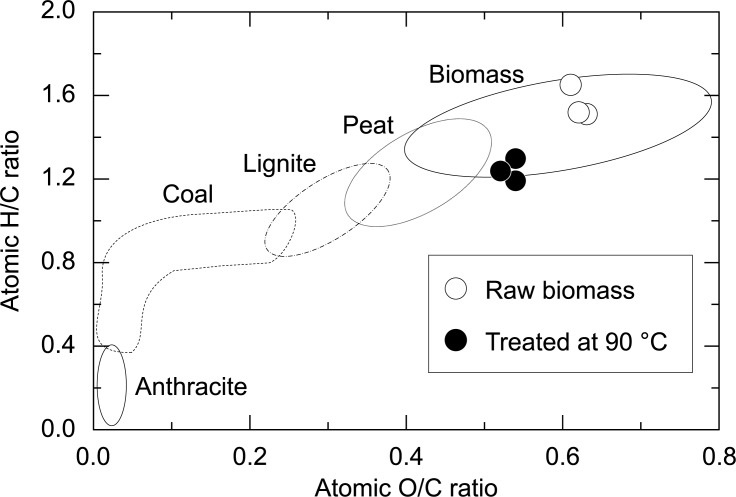
van Krevelen diagram of raw manure and manure treated at 90 °C.

The physicochemical properties of the raw and treated biomass are tabulated in Table 1. Elemental analysis revealed that H and O in the raw biomass were reduced, whereas the carbon content was essentially unchanged. The reduction rate of C, H and O was 27.5%, 41.5%, and 36.8%, respectively. The HHV of the product decreased slightly compared to the raw biomass. We determined the severity of this process by plotting the van Krevelen diagram (atomic H/C ratio versus the atomic O/C ratio, Fig 6). Manure heated at 90 °C moved from the biomass zone to the boundary region between biomass and peat.

## Discussion

### Biomass decomposition during a low temperature process

The results demonstrated that manure heated at 90 °C for 49 days was transformed into a peat-like material. Biomass decomposition accompanied by the evolution of VFAs, CO, and CO_2_ was observed under an oxidative environment and conversion stopped when the biomass was dehydrated, indicating that biomass is decomposed not by a thermal process but rather by an oxidative process governed by the presence of water in the biomass.

**Fig 7:**
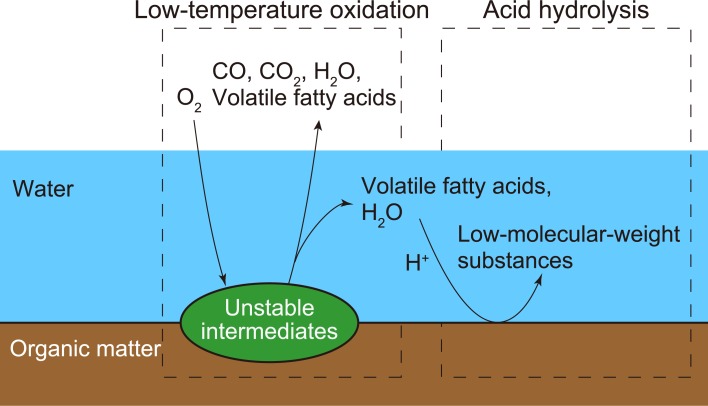
Biomass decomposition below 100 °C.

Decomposition is believed to involve both oxidative decomposition and acid hydrolysis. The decomposition products of biomass include VFAs that release hydrogen ions (Fig 7). Hemicellulose and lignin are acid-hydrolyzed even at temperatures as low as 90 °C [19,20]. Therefore, biomass decomposition could be caused by the action of VFAs generated by oxidative decomposition.

Based on the present findings, we believe that the process of biomass decomposition at temperatures below 100 °C is as illustrated in Fig 7. In this process, biomass undergoes oxidation and acid hydrolysis by the action of water and oxygen while releasing CO, CO_2_, H_2_O, and VFAs such as acetic, propionic, isobutyric, and isovaleric acids. It should be noted that H_2_O, CO_2_, and acetic acid are released as the major volatile products in dry torrefaction [21,22], suggesting that the biomass in the present study underwent a decomposition process at 90 °C in the presence of oxygen and water similar to the torrefaction process, despite the lower temperature.

### Possibility of microbial involvement in biomass decomposition

The production of a peat-like material under an air environment may allow microbial reactions such as composting. However, microbial activity is unlikely for the following reasons. First, microbial activity significantly decreases above 63 °C due to increasing thermal inactivation [23]. Indeed, the temperature of a compost pile is typically around 75 °C and rarely above 90 °C, indicating that even thermophiles are generally inactivated above this temperature. In addition, microbial activity is affected by the moisture content and decreases below 35%wb [24,25]. The current study observed the maximum rate of gas evolution at a critical moisture content (around 12%wb) and this does not support microbial involvement. Second, the condensate liquids were strongly acidic and far below the optimum pH values (pH: 5.5–8) for composting [26]. Although the pH value decreases initially during composting, it does not drop below 5 [17,26–28]. Thus, it is reasonable to assume that biomass decomposition at 90 °C is not caused by microbial reactions but rather by chemical reactions.

### Feasibility of the proposed methodology

The physicochemical properties of the product showed that biomass quality was not improved significantly despite our attempt. Calorific value is one criterion for evaluating fuel quality and is often increased through thermochemical conversion processes. However, the proposed methodology did not improve the calorific value due to (i) the insufficient oxygen removal, (ii) a decrease in hydrogen, and (iii) an increase in ash and nitrogen. These factors were closely related to the low temperature used and carbon loss by oxidation. In addition, the process requires a longer period of time compared to conventional thermochemical conversion technologies. Taken together, the proposed technique may be inappropriate as a general biomass valorization technology but could be applicable in certain situations for stabilizing biomass. For example, the process could be conducted using waste heat from the industrial sector. In the U.S., manufacturing processes release low-grade waste heat at temperatures below 230 °C [29]. Although this waste heat is difficult to harness as a heat source for torrefaction, its temperature is suitable for the proposed technique and would allow biomass to be stabilized through mild degradation and adequate drying without additional energy input, allowing the processed biomass to be utilized as a renewable source.

Some waste biomass, sewage sludge and manure may contain pathogenic microbes which would need to be killed during the process. This is typically achieved by exposing biomass to a certain temperature for a given duration (e.g., 55 to 65 °C for 24 hours to 3 days) [30]. In addition, decreasing the moisture content is effective because all microbial growth slows and then ceases at less than 25% water [30]. In the proposed technique, biomass is treated at temperatures much higher than 55 to 65 °C and is thoroughly dehydrated and thus the processed biomass is likely microbe-free.

## Conclusions

An oxidative environment and the water in biomass promote biomass decomposition at 90 °C. The drying and decomposition of biomass proceed simultaneously, and decomposition continues until the water has been removed. Biomass decomposition is accomplished not by composting but rather by low-temperature oxidation and acid hydrolysis, with the release of CO, CO_2_, H_2_O and VFAs. Although the severity of the process is relatively weak due to the low temperature used, the biomass product is nonetheless a peat-like material. The proposed technique can stabilize waste biomass with lower energy consumption than current approaches, increasing the opportunity to supply bioenergy from biomass.

## Supporting information

S1 Fig GC-MS chromatograph of the condensate liquid. (EPS)
